# Effectiveness of Impregnated Central Venous Catheters on Catheter-Related Bloodstream Infection in Pediatrics

**DOI:** 10.3389/fped.2022.795019

**Published:** 2022-03-03

**Authors:** Zhengrong Deng, Jiangwei Qin, Huanbin Sun, Furong Xv, Yimei Ma

**Affiliations:** ^1^Department of Pediatrics, West China Second University Hospital, Sichuan University, Chengdu, China; ^2^Key Laboratory of Birth Defects and Related Diseases of Women and Children (Sichuan University), Ministry of Education, Chengdu, China; ^3^West China Hospital, Sichuan University, Chengdu, China; ^4^West China School of Basic Medical Sciences and Forensic Medicine, Sichuan University, Chengdu, China; ^5^Wuyuzhang Honors College, Sichuan University, Chengdu, China

**Keywords:** antibiotics, impregnations, central venous catheters, bloodstream infection, pediatrics

## Abstract

**Background:**

The efficacy and safety of impregnated central venous catheters (CVCs) in pediatrics remain controversial. The purpose of this study was to evaluate the efficacy of impregnations for the prevention of catheter-related bloodstream infection (CRBSI).

**Methods:**

We searched the following five electronic databases: Medline, PubMed, Cochrane, Embase, and the Web of Science for randomized controlled trials (RCTs) up to March 2021. Pooled risk ratios (RRs) with 95% confidence intervals (CIs) were calculated using a fixed-effects model. Assessment of publication biases was evaluated by Egger's test. Heterogeneity between studies was assessed based on the chi-square test and *I*^2^ statistics, and sensitivity analysis and subgroup analysis were also performed.

**Results:**

A total of six RCTs with 3,091 patients were included. Impregnated CVCs provided significant benefits in reducing the risk of CRBSI (RR = 0.41, 95% CI: 0.26–0.66) in pediatric patients, especially in the pediatric group. No publication bias was observed in the Egger test for the risk of CRBSI. Drug type is a source of heterogeneity.

**Conclusion:**

Antimicrobial-impregnated CVCs are beneficial to prevent CVC-related complications in pediatrics.

## Introduction

Central venous catheters (CVCs) are clinically important in the treatment of venous pressure monitoring, infusion of drugs, and nutrient fluid supplementation (1). However, the widespread use of CVCs has been shown to increase the risk of developing catheter-related bloodstream infection (CRBSI) (2). It has been reported that CRBSIs can occur in 13–20% of catheterized newborns by the Centers for Disease Control and Prevention (3) and that 1 in 10 children with a central venous catheter develops a central line-associated bloodstream infection (4). It is well-known that CRBSIs increase children's mortality, exposure to antibiotics, direct cost, and length of stay in the hospital (5, 6).

The prevention strategies of CRBSI being recommended include highlighting hand hygiene (7), maximal sterile barrier precautions (8), and avoidance of the femoral site for catheter insertion (9). However, with each strategy having potential clinical advantages, it is unclear whether there are better clinical outcomes than the other treatment regimens. The most promising approach is the use of impregnated CVCs, which is recommended by the US and UK national guidelines for patients at high risks of infection (10). Impregnated CVC effectiveness comparing standard, non-impregnated CVCs has been provided in previous adult systematic analyses (11–13). Antimicrobial agents such as chlorhexidine-, minocycline-rifampicin-, and silver sulfadiazine-impregnated (14) CVCs have shown beneficial outcomes in preventing CRBSI in adults. Impregnated central venous catheters, however, have not enough evidence for their effect on reducing the rates of CRBSI in pediatrics. Furthermore, several new RCTs on this issue have been reported.

Given this background, we performed a meta-analysis study of available studies to evaluate the efficacy of central venous catheters impregnated with heparin and antimicrobial agents in comparison with standard and non-impregnated catheters on the prevention of CRBSI.

## Materials and Methods

We conducted the meta-analysis according to the Preferred Reporting Items for Systematic Review and Meta-Analysis Protocol (PRISMAP) (15).

### Search Strategy and Selection Criteria

The search was performed up to March 2021 in PubMed, Medline, Embase, the Cochrane Central Register of Controlled Trials, and the Web of Science using MeSH terms and text words for published clinical trials, and the references of relevant meta-analyses and other internet sources for unpublished clinical trials were also considered. The following terms were adjusted according to different search rules in each database: “impregnated,” “catheters,” “pediatrics,” “bloodstream,” and “infections.” We also searched ClinicalTrials.gov and the WHO International Clinical Trials Registry Platform for unpublished, planned, or ongoing trial reports. In addition, we contacted the authors of the included RCTs to obtain additional data if necessary. All studies were imported into EndNote for duplication exclusion.

### Criteria for Included Studies

All potential studies were screened based on the PICOS (population, intervention, comparator, outcome, and study design) standard. We defined the terms as follows: (1) Population: all patients with vascular catheters inserted for an expected duration of more than 72 h under the age of 18; (2) Intervention: the intervention method was impregnation-treated CVCs with no limitation of drug types, and the control group refers to conventional, non-impregnated CVCs; (3) Comparison: the objective was to compare the efficacy of impregnated CVCs and non-impregnated CVCs in the prevention of CRBSI in children; (4) Outcomes: the outcomes included the incidence of CRBSIs and thrombosis; (5) Study design: all study designs were prospective RCTs.

### Outcome Measures

The primary outcome in this meta-analysis was the incidence of CRBSIs. Furthermore, we also considered the incidence of thrombosis as the secondary outcome with incomplete data among the included studies.

### Data Extraction

Two reviewers (Zhengrong Deng and Jiangwei Qin) independently evaluated the titles, abstracts, and full texts of identified studies using predefined criteria and extracted study characteristics and outcome data for each study, such as first author name, publication year, country, age of population, numbers of participants, interventions, clinical signs, and brief conclusion, with all the data cross-checked by other authors. Any disagreements were resolved to a consensus by team discussion.

### Quality Assessment

The risk of bias of the included RCTs was evaluated using the Cochrane Risk of Bias Scale independently using the following criteria: (1) random sequence generation (selection bias), (2) allocation concealment (selection bias), (3) blinding of participants and personnel (performance bias), (4) incomplete outcome data (attention bias), (5) selective reporting (reporting bias), and (6) other sources of bias. For each criterion, studies were classified as having a low, high, or unclear risk of bias.

### Statistical Analyses

The analyses were performed in Review Manager (Version 5.3) and R (Version 4.04). The incidence of CRBSI and thrombosis are binary outcomes, so risk ratios (RRs) with 95% confidence intervals (95% CIs) were calculated using the Mantel–Haenszel method. If there was no statistically significant heterogeneity among the studies (*I*^2^ <50%, *p* > 0.05), a fixed-effects model was used; otherwise, a random-effects model was considered. We set *p* <0.05 as statistically significant for hypothesis testing. Heterogeneity between studies was also assessed based on the chi-square test by *I*^2^ statistics. Assessment of publication biases was evaluated by Egger regression. Sensitivity analysis was conducted to identify the influence of each study on the synthesized results. *p* <0.05 was considered statistically significant. Subgroup analyses based on impregnation drug type and between neonates (age ≤ 28 days) and children (28 days < age ≤ 18 years) were undertaken to determine the source of heterogeneity. Furthermore, meta-regression analyses were used to detect whether other variables, such as publication year and bloodstream infection (BSI) reported, caused heterogeneity to some extent.

## Results

### Study Selection and Data Extraction

**Figure 1:**
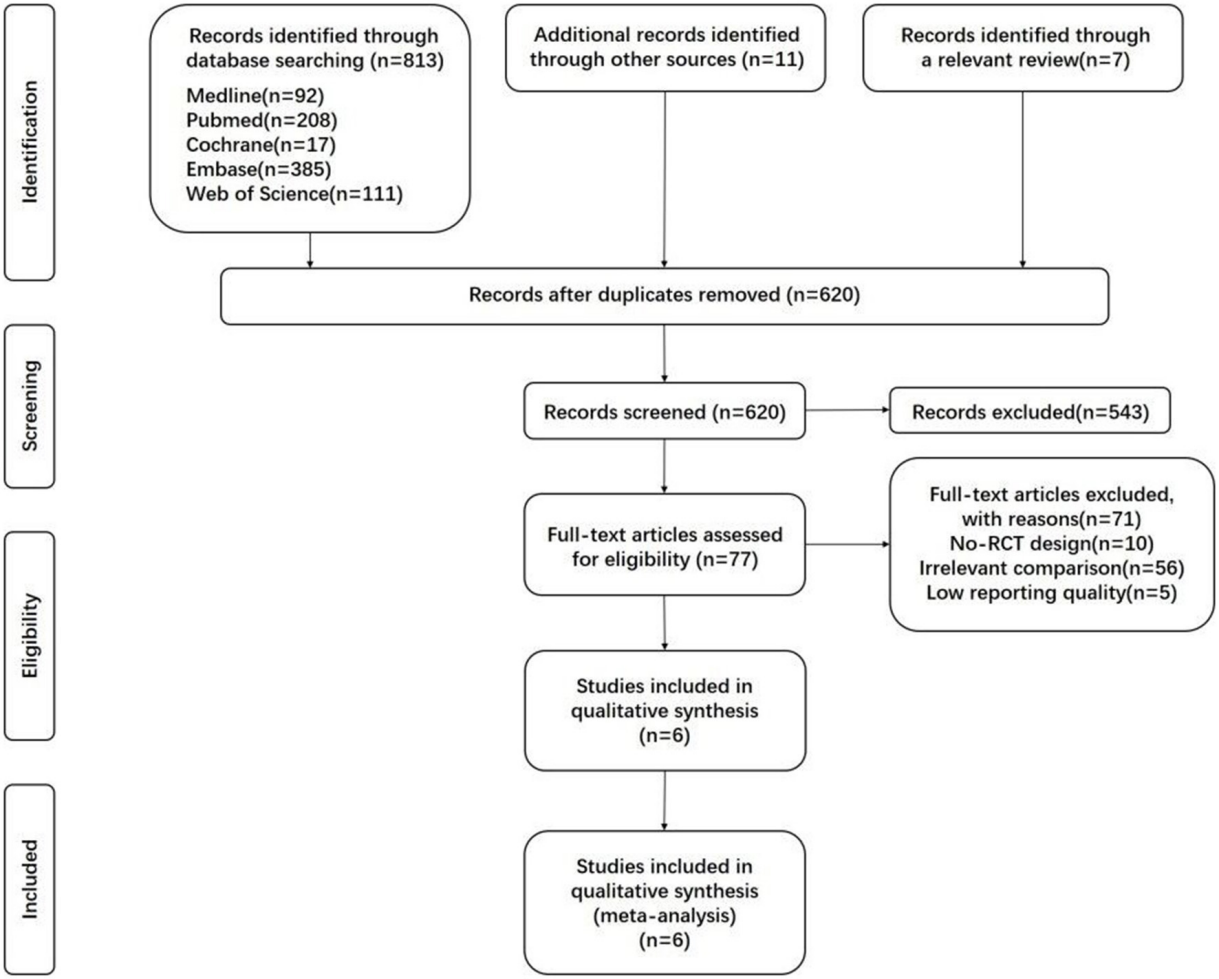
PRISMA flowchart for study selection.

The search identified 813 records, of which 229 duplicates were removed. In addition, 18 other studies were included from the reference list of a relevant review and other sources. Of the remaining 620 articles, 77 were relevant after inspecting the titles. During the full-text screening, 71 articles were excluded due to No-RCT design, irrelevant comparison, or low reporting quality, leaving six articles to be included in this meta-analysis. These studies included a total of 3,091 patients, with 1,781 patients for impregnated CVC intervention and 1,310 patients for standard CVC intervention. Figure 1 presents the PRISMA flowchart for study selection. Supplementary Table S1 (16–21) presents the characteristics of each study. For the type of CVC, other details were reported among the included RCTs, as described in Supplementary Table S2. There would be little probability that heterogeneity originated from catheter materials because they are all composed of polyurethane. Conclusions suggested that three studies detected that antimicrobial-impregnated CVC did not provide a protective effect of CRBSI, while three other studies found that antimicrobial-impregnated CVC significantly reduced the risk of CRBSI.

### Bias Evaluation

**Figure 2:**
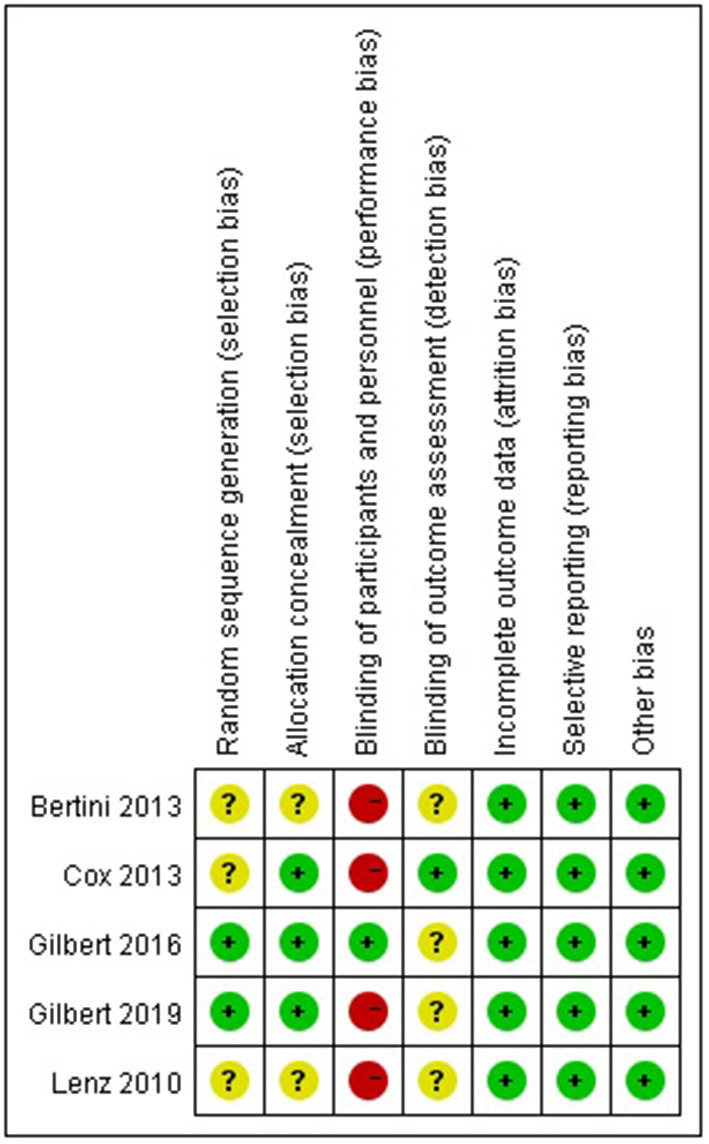
Risk of bias summary.

The results of the quality assessment of the five RCTs included are presented in Figure 2. The outcomes indicated the risks of bias for each included study. Briefly, all included RCTs mentioned randomization in their reports, but only two RCTs reported the methods to generate random sequences. Two studies failed to report the methods to perform allocation concealment. Four studies were rated as having a high risk of performance bias, as they were unable to blind the personnel or participants to the intervention allocated. One RCTs reported a blind design during the outcome assessment. No other kinds of biases were found.

### Primary Outcome

#### The Rate of CRBSIs

**Figure 3:**
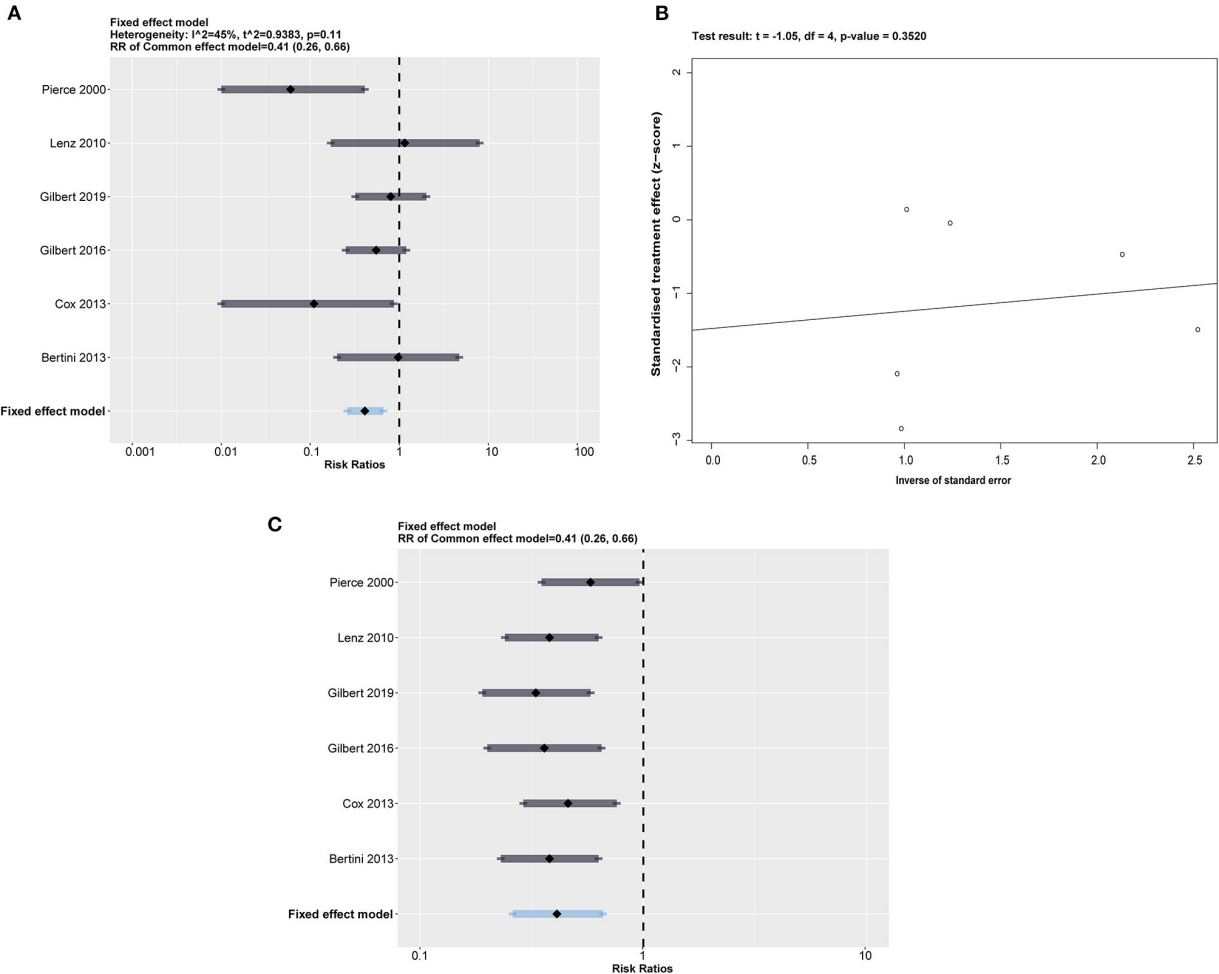
**(A)** Forest plot of the risk of CRBSI for impregnated catheters. All impregnated medicines were calculated together, with a summary RR = 0.41 (0.26, 0.66) under a fixed effect model. **(B)** Egger's test plot shows an acceptable *p* > 0.3520. **(C)** The sensitivity analysis performed by eliminating one included study in a time represents a relatively robust result.

The impregnated CVCs had lower rates than the control group in the risk of CRBSI (RR = 0.41; 95% CI 0.26 to 0.66; *p* = 0.0002, weighted based on sample size). A fixed effects model was used for the meta-analysis because of moderate heterogeneity (*I*^2^ = 45%, *p* = 0.11) (Figure 3A). We also obtained *Q* = 9.04. *I*^2^ and *Q* suggest the appearance of heterogeneity amounting equally with random error.

#### Publication Bias

We performed Egger's test of funnel plot asymmetry on the risk of CRBSI in Figure 3B, which indicated that no publication bias was detected (*t* = −1.05, *p* = 0.3520 > 0.05).

### Sensitivity Analyses

Considering the moderate heterogeneity in the synthetic result, we conducted a sensitivity analysis by excluding 1 study at a time to estimate whether the results could have been affected markedly by a single study (Figure 3C). The results showed that Pierce et al. (16) accounted for a significant percentage of the bias of effect size. However, we found that the overall results were not changed by exclusion of any included RCTs. Considering that Pierce et al. (16) is one of only two studies using heparin-bonded CVCs, we suspected that catheter type is a potential source of heterogeneity. Heparin has been recognized as a valuable antithrombotic drug without direct antibacterial effects compared with chlorhexidine antiseptics and silver ions (22). Pierce et al. (16) suggested that heparin may prevent CRBSI by reducing thrombosis and bacterial colonization, acting as an indirect action on CRBSI.

### Subgroup Analysis

**Figure 4:**
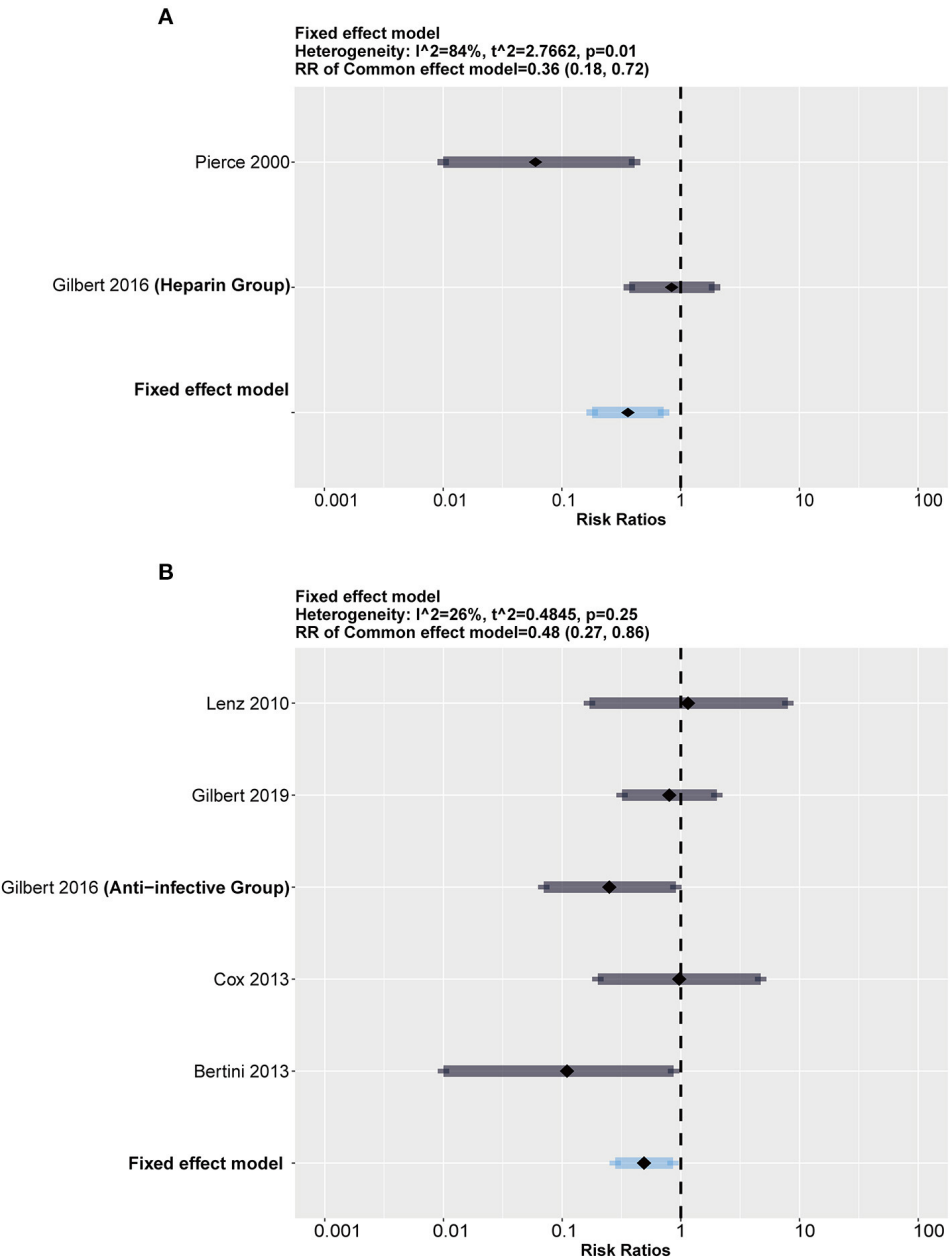
Subgroup analysis between heparin and antimicrobial drugs. The forest showed a significant difference in both groups but a much higher heterogeneity in the heparin group than in the antimicrobial group. **(A)** Forest plot in heparin group (compared with standard). **(B)** Forest plot in antimicrobial group (compared with standard).

To test our assumption, we conducted subgroup analysis stratified by heparin-impregnated CVCs and antimicrobial-impregnated catheters. As Figure 4 shows, the antimicrobial-impregnated CVC group presented lower heterogeneity (*I*^2^ = 26%, *p* = 0.25), while impregnated heparin showed higher heterogeneity (*I*^2^ = 84%, *p* = 0.01), indicating that the efficiency of heparin impregnated on CRBSI was unstable. In addition, it showed that the antimicrobial-impregnated catheter provided more benefits (RR = 0.48; 95% CI: 0.27–0.86, weighted based on sample size) in reducing the risk of CRBSI among the included RCTs compared to the heparin-impregnated CVCs.

**Figure 5:**
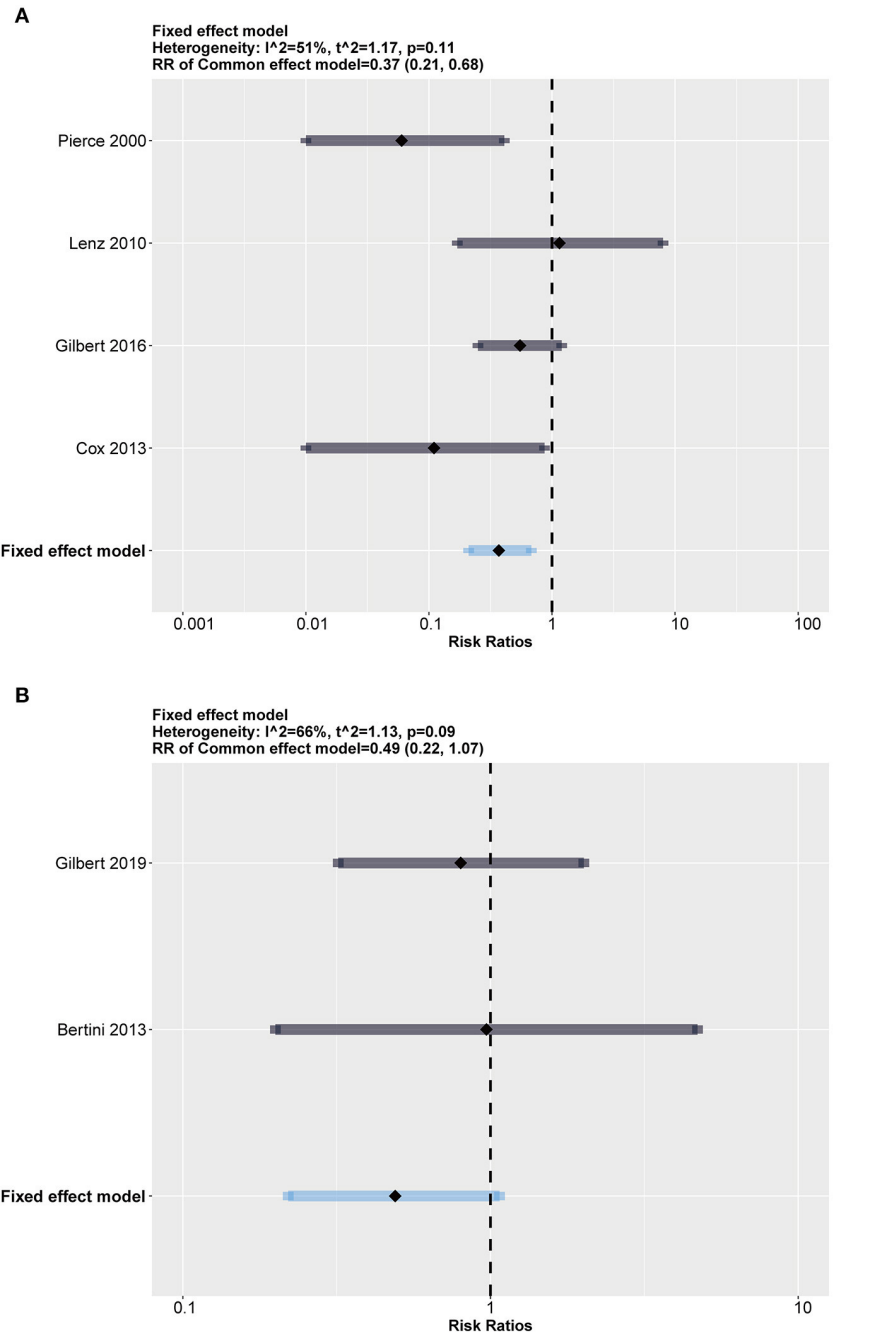
Subgroup analysis between neonates (age ≤ 28 days) and children (28 days < age ≤ 18 years). Both groups show high heterogeneity. **(A)** Forest plot in children (28 days < age ≤ 18 years) group (compared with standard). **(B)** Forest plot in neonates (age ≤ 28 days) group (compared with standard).

We also added an exploratory subgroup analysis regarding the differences in infection rates between neonates (age ≤ 28 days) and children (28 days < age ≤ 18 years) for discrepant immune system development. The results shown in Figure 5 indicate that both groups had a relatively large scale of heterogeneity (*I*^2^ = 51%, *p* = 0.11 in the neonate group; *I*^2^ = 66%, *p* = 0.09 in the children group). It should be noted that although results with heterogeneity should be considered conservatively, impregnated CVCs seemed to protect children from CRBSI with an RR = 0.37 (95% CI: 0.21–0.68, weighted based on sample size).

### Secondary Outcome

#### The Rate of Thrombosis

**Figure 6:**
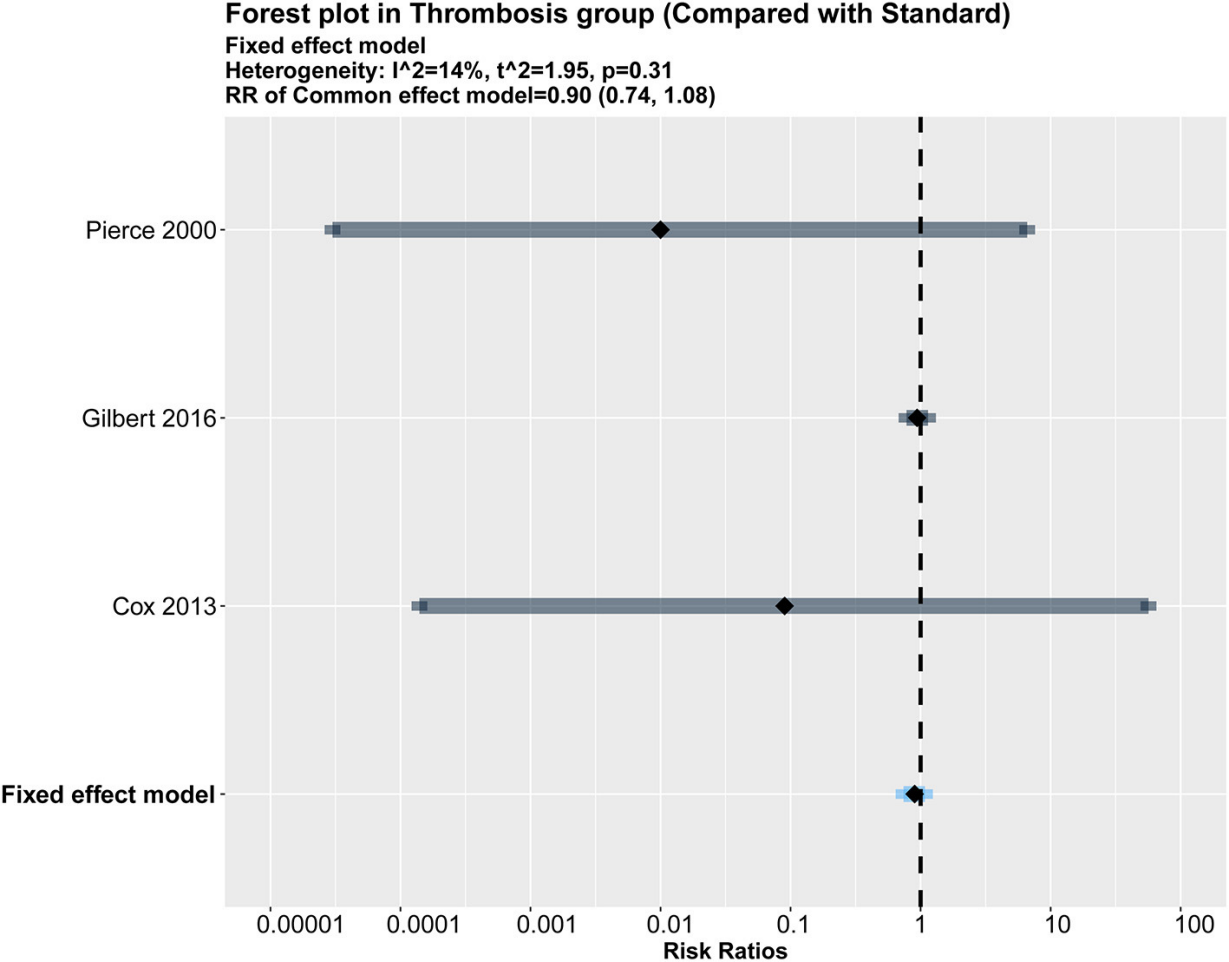
Forest plot of the risk of thrombosis for impregnated catheters.

A meta-analysis of three studies revealed no significant differences in the rate of thrombosis between impregnated and standard CVCs (16, 19, 20) (RR = 0.90, 95% CI: 0.74–1.08, *p* = 0.253). A fixed model was used because no heterogeneity was noted among the studies (*I*^2^ = 14%, *p* = 0.31). The results are presented in Figure 6.

## Discussion

We exhaustively collected RCTs related to impregnated CVCs in pediatrics, which included agents such as heparin, AgION, minocycline, miconazole, and rifampicin. Heparin is a natural anticoagulant substance in animals, and its anti-infective function was suspected to be related to preventing bacterial colonization through beneficial hemodynamic function. However, as we described in the subgroup analysis in Figure 4, the clinical anti-infective effect is unstable and requires other studies for more convincing results. AgION, as a patented silver compound, has been reported to be effective in adult treatment (23) because of the bactericidal activity of silver ions. Minocycline, a second-generation, semisynthetic tetracycline, can effectively reduce the risk of CRBSI in pediatrics due to its antibiotic properties against both Gram-positive and Gram-negative bacteria (20). Miconazole and rifampicin combination is a common antimicrobial combination impregnated strategy of catheters, which was estimated to reduce infections from 5 to 0% to save $500,000 per 850 catheters used (24). The use of the above antimicrobial agents can theoretically reduce the risk of relevant infections, as reported in our results.

It has been reported that pediatric intensive care units (PICUs) have one of the highest rates of acquired bloodstream infections in hospitals, especially after cardiac surgery (25), with central venous catheters being a frequent source. PICU admission for patients with nosocomial primary BSI would have ~6 times the direct cost compared with patients without BSI, as reported by St Louis Children's Hospital in 2005 (26).

To date, much new evidence has basically unified the perspective that impregnated CVCs are recommended for adults to reduce bloodstream infections. Chong et al. conducted a network meta-analysis including 60 studies with 17,255 catheters and investigated the effects of 14 impregnation drugs on CRBSI and catheter colonization. Significant CRBSI reduction was associated with minocycline-rifampicin (RR = 0.29 [95% CI: 0.16–0.52]) and silver (RR = 0.57 [95% CI: 0.38–0.86]) impregnations compared to no impregnation. For colonization, miconazole-rifampicin (RR = 0.14 [95% CI: 0.05–0.36]), 5-fluorouracil (RR = 0.34 [95% CI: 0.14–0.82]), and chlorhexidine-silver sulfadiazine (RR = 0.60 [95% CI: 0.50–0.72]) impregnations showed significant decreases compared with non-impregnation (11). Wei et al. (12) reported that chlorhexidine-impregnated dressings provided significant benefits in reducing the risk of catheter colonization (OR = 0.46, 95% CI: 0.36–0.58) and CRBSI (OR = 0.60, 95% CI: 0.42–0.85). Huang et al. (27) analyzed chlorhexidine-, heparin-, and antimicrobial-impregnated CVCs in a systematic review and gave a positive conclusion. All of the above meta-analyses indicate positive outcomes on impregnations, including minocycline, rifampicin, silver ion, chlorhexidine, and heparin, in the control of CRBSI in adults. In pediatrics, however, there are no such large simple size systematic analyses to prove whether impregnated CVCs are effective or not due to a lack of enough RCTs. It is necessary to combine existing studies related to children to complement clinical guidelines.

Our systematic review and meta-analysis provided the most recent and comprehensive analysis of the efficiency of impregnated CVCs for the prevention of CRBSI in pediatrics. With six RCTs included, the results of this meta-analysis suggest that the use of impregnated CVCs is beneficial to reduce the risk of CRBSI for patients with CVC and is an effective anti-infection strategy in preventing CRBSI (RR = 0.41; 95% CI: 0.26–0.66; *p* = 0.0002). Our further subgroup analysis indicated that the use of antimicrobial drug-impregnated CVCs had a significantly lower risk of CRBSI (RR = 0.48) than the conventional catheter group in the pediatric population. In addition, after considering differences between neonates and children, the age cutoff at 28 days supported a prominent prevention effect in the pediatric group (28 days < age ≤ 18 years). Our results support the benefits antimicrobial drug-impregnated CVCs would have in pediatric patients, especially in children.

The results were consistent with previous findings (28), but with more RCTs included for synthesized analysis, our results do provide more strength in increasing the statistical effectiveness. The newly included study by Gilbert et al. (21) represented a better quality in both random sequence generation and allocation concealment, also with a higher weight for larger sample sizes. Its risk ratio (RR = 0.80, 95% CI: 0.31–2.01) exceeded the combined risk ratio in Wu et al. (28) (RR = 0.28, 95% CI: 0.13 and 1.09), but this RCT with high quality led to a smaller confidence interval and a beneficial effect of impregnated CVCs.

There are several limitations that need to be considered in this meta-analysis. First, the main limitation is that our meta-analysis only included six eligible RCTs, which were relatively small in the establishment of prognostic value. Second, the antimicrobial concentration used for impregnated CVC is uncertain. Third, we only performed subgroup analyses stratified by impregnation type and age population but not by the frequency of dressing change and the duration of catheterization. We attempted to conduct subgroup analysis according to the frequency of dressing change, but the data among the included RCTs were not fully available. Finally, due to data limitations, the role of heparin-impregnated CVCs remains unclear, and future studies evaluating the efficiency of heparin-impregnated CVCs for preventing CRBSI in pediatric patients are warranted.

## Conclusion

Our results indicate that antimicrobial-impregnated CVCs have a beneficial effect on decreasing the CRBSI risk in children aged under 18 years compared with non-impregnated standard CVCs, especially in children (28 days < age ≤ 18 years).

## Data Availability Statement

The datasets generated for this study are available on request to the corresponding author.

## Author Contributions

YM, ZD, and JQ: contributed to the study conception, design, data acquisition, and interpretation.YM, ZD, JQ, HS, and FX: contributed to the data analysis and drafted the manuscript. All authors contributed to the critical revision of the manuscript and approved the final version.

## Funding

This study was supported by a grant from the West China Second University Hospital of Sichuan University, KX144, which funded the design of the study and collection, analysis, and interpretation of data and the writing of the manuscript.

## Conflict of Interest

The authors declare that the research was conducted in the absence of any commercial or financial relationships that could be construed as a potential conflict of interest.

## Publisher's Note

All claims expressed in this article are solely those of the authors and do not necessarily represent those of their affiliated organizations, or those of the publisher, the editors and the reviewers. Any product that may be evaluated in this article, or claim that may be made by its manufacturer, is not guaranteed or endorsed by the publisher.

## Supplementary Material

Supplementary Table S1 Characteristics of the included studies. CICU, cardiac intensive care unit; PICC, peripheral inserted central venous catheter.

Supplementary Table S2 Details of catheter use in six included studies. We collected detailed information on the catheters used in each study. Sizes differed in accordance with children's age, weight, and health state, as well for lumen numbers. Materials were all the same among six studies after scrutinizing manufacturers of catheters.
