# Supplementary material for: Effectiveness of Impregnated Central Venous Catheters on Catheter-Related Bloodstream Infection in Pediatrics
Source: Front Pediatr. 2022 Mar 3;10:795019. doi: 10.3389/fped.2022.795019 (PMC8927082; doi:10.3389/fped.2022.795019)
Supplement: Supplementary Table S1 — Characteristics of the included studies. CICU, cardiac intensive care unit; PICC, peripheral inserted central venous catheter. [file Table_1.DOCX]

**Table S1. The characteristics of included studies**

| **Author(Year)** | **Country** | **Population** | **Numbers of participants (Interventions/Control)** | **Interventions** | **Clinical signs** | **Randomization concealed; clinicians blinded** | **Conclusion** |
| --- | --- | --- | --- | --- | --- | --- | --- |
|  |  |  |  |  |  |  |  |
| **Pierce (2000)** | USA | Aged 0-16 years, admitted to intensive care unit, and needing a central venous line | 200(97/103) | Central venous line; heparin-bonded | Yes + at removal | Adequate, Blinded | Study shows a significant reduction in the incidence of infection and thrombosis associated with the use of heparin-impregnated CVCs |
|  |  |  |  |  |  |  |  |
| **Lenz (2010)** | Argentina | Ages 1 day to 1 year, admitted to CICU | 172(80/92) | CVC; antibiotic impregnated | Yes + at removal | NS, NS | Antibiotics-impregnated CVC would not provide a protective effect of colonization with respect to standard CVC. |
|  |  |  |  |  |  |  |  |
| **Bertini (2013)** | Italy | Ages 1 day to 30weeks | 86(45/41) | UVC; AgION impregnated; | Yes + at removal | NS, Not Blinded | The use of an AgION-impregnated UVC is effective in decreasing the risk of developing a CRBSI in preterm infants. |
| **Cox (2013)** | USA | Ages 1 day to 18 years | 287(146/141) | CVC;;miconazole and rifampicin-impregnated | Yes + NS | Adequate, Not Blinded | No difference was observed in infectious outcomes between impregnated and standard central venous line catheters. |
|  |  |  |  |  |  |  |  |
| **Gilbert (2016)** | UK | Ages 1 day to 16 years, admitted or being prepared for admission to a participating CICU | 1485(983/502) | CVC; antibiotic impregnated; heparin bonded | Yes + at removal | Adequate, Blinded | Antibiotic-impregnated central venous catheters significantly reduced the risk of bloodstream infections compared with standard and heparin central venous catheters. |
|  |  |  |  |  |  |  |  |
| **Gilbert (2019)** | UK | Ages 1 day to 32 weeks | 861(430/431) | PICC; miconazole and rifampicin-impregnated | Yes + at removal | Adequate, Blinded | No evidence of benefit or harm is associated with miconazole and rifampicin impregnated PICCs compared with standard PICCs for newborn babies. |

**Abbreviation: BSI Blood Stream Infection; CICU Cardiac Intensive Care Unit; PICC Peripheral Inserted Central Venous Catheter**
