# Supplementary material for: Effectiveness of Impregnated Central Venous Catheters on Catheter-Related Bloodstream Infection in Pediatrics
Source: Front Pediatr. 2022 Mar 3;10:795019. doi: 10.3389/fped.2022.795019 (PMC8927082; doi:10.3389/fped.2022.795019)
Supplement: Supplementary Table S2 — Details of catheter use in six included studies. We collected detailed information on the catheters used in each study. Sizes differed in accordance with children's age, weight, and health state, as well for lumen numbers. Materials were all the same among six studies after scrutinizing manufacturers of catheters. [file Table_2.DOCX]

**Table S2. The characteristics of included studies**

| **Research** | **Catheter type** | | | | **Manufacturer** | **Products introduction page** |
| --- | --- | --- | --- | --- | --- | --- |
|  | **Sizes** | | **Lumen** | **Materials** |  |  |
|  | **Diameter** | **Length** |  |  |  |  |
| **Pierce (2000)** | 4 Fr; 5 Fr | 5 cm; 8 cm; 15 cm | double lumen; trible lumen | Polyurethane | Cook Critical Care | https://www.cookmedical.com/products/ |
| **Lenz (2010)** | Diameter not stated | 13 cm | double lumen | Polyurethane | Teleflex Medical Oem | https://www.teleflex.com/la/en/product-areas/vascular-access/central-venous-catheters/index.html |
| **Bertini（2013）** | 4 Fr; 5 Fr | Length not stated | Lumen not stated | Polyurethane | Vygon | https://www.vygon.com/catalog/lifecath_1700_00219127 |
|  | 3.5 Fr; 5 Fr |  |  | Polyurethane | Kendall | https://www.medtronic.com/covidien/en-us/products/dialysis-access/peritoneal/argyle-catheters.html |
| **Cox（2013）** | 4 Fr; 5 Fr | 8 cm; 12 cm | double lumen | Polyurethane | Cook Critical Care | https://www.cookmedical.com/products/ |
| **Gilbert (2016)** | 4 Fr; 5 Fr ; 7 Fr | Length not stated | double lumen; trible lumen | Polyurethane | Cook Critical Care | https://www.cookmedical.com/products/ |
| **Gilbert (2019)** | 1 Fr | 20 cm | Single lumen | Polyurethane | Vygon | https://www.vygon.com/catalog/premistar_1496_00626120 |

**Abbreviation: BSI Blood Stream Infection; CICU Cardiac Intensive Care Unit; PICC Peripheral Inserted Central Venous Catheter**
